# Academy of Sciences, Paris—Experimental Physiology

**Published:** 1868-08-01

**Authors:** 


CHICAGO MEDICAL JOURNAL.

*Vol. XXV.*--*AUGUST* 1, 1868.—*No.* 15.

### ACADEMY OF SCIENCES, PARIS.—EXPERIMENTAL PHYSIOLOGY.

### AWARD OF THE MONTYON PRIZE.

(Commissioners: MM. Longet, Milne-Edwards, Ch. Robin, de Quatrefages, Claude Bernard, Reporter.)

TRANSLATED BY WALTER HAY, M.D., ASSOCIATE EDITOR CHICAGO MEDICAL JOURNAL.

EVERY problem of experimental physiology is generally so complex, that it would be delusion or rashness on the part of any single author to take upon himself alone its solution and exhaustion. These subjects, ordinarily, are illuminated only by a series of associated efforts, in proportion as our means of investigation are perfected, and as experimental analysis penetrates more profoundly into the mechanism of phenomena. These remarks apply with perfect accuracy to the difficult question of the innervation of the heart by the spinal cord, which has already been the subject of the successive investigations of a great number of distinguished experimenters.

At the end of the last century, Haller* still considered the movements of the heart as being independent of all nervous influence. He founded his opinion upon the possibility of the

* Haller, Memoire sur l'irritabilité, 1777.

continuation of the circulation in an animal deprived of its brain, and upon this fact that a heart torn from the chest may beat and contract.

It was at the commencement of the present century that Le Gallois* discovered that the influence of the spinal cord is necessary to the maintenance of the pulsations of the heart, and he demonstrated experimentally that the destruction, either total or partial, of this nervous centre prevented the regular continuation of the circulation of the blood, even with the aid of artificial respiration.

Still later, Magendie, and a member of this commission† made use, for the first time, of a hæmometer, or cardiometer, with the intention to investigate and render more evident the modifications effected in the movements of the heart by the irritation of the spinal cord and of the nerves which originate therein. These experiments established the two following new results :

1st. The irritation of the sensitive spinal nerves induces a constant modification in the pressure of the blood, and an alteration in the pulsations of the heart.

2d. This action, which is of a reflex character, is not transmitted to the heart by the pneumogastric nerves, for it manifests itself even after the section of these nerves in the middle region of the neck.

In 1863, M. de Bezold‡ instituted experiments designed to throw light upon the influences which the spinal cord exercises upon the heart. This author established in his work two important facts. He showed first that section of the spinal cord between the occiput and the atlas, produced a very considerable diminution in the pressure of the blood in the large arteries, and that induces a retardation in the pulsations of the heart. He proved, moreover, that irritation of the cord below (posterior

* Œuvres de Le Gallois, édition de Pariset, t. I.: Experience sur le principe de la vie, et sur les mouvements du cœur.

† Comptes rendûs des séances de l'Académie des Sciences, t. XXIV, p. 1130.—Claude Bernard. Lecons sur la physiologie du systéme nerveux, t. I, page 271-275, 1858.

‡ Albert von Bezold, Untersŭchungen über die Innervation des Herzens, 1863.

to) the section re-established the pressure of the blood, and caused it to ascend even above the normal amount, at the same time producing an acceleration in the cardiac pulsations. M. de Bezold believes that he has demonstrated by these last experiments, that the spinal cord reacts directly upon the movements of the heart, and it is at this conclusion, indeed, that he stopped.

But soon, MM. Ludwig and Thiry* combatted this opinion, by interpreting, entirely differently, the facts, otherwise exact, established by M. Bezold; and MM. Ludwig and Thiry deny all direct nervous action upon the heart, relying upon the fact that the spinal cord separated from the brain always exercises its influence upon the pressure of the blood even when all the cardiac nerves, which unite the heart with the cord, have been destroyed by the galvano-caustic method. They proceed even to prove that it is not necessary to excite the spinal cord in order to obtain the results already indicated, for simple compression of the aorta by restricting the area of the circulation may determine an augmentation in the manometric pressure of the blood. As to the acceleration of the pulsations of the heart, which coincide here with the increase to the resistance to the circulation, it will be perceived later that it becomes necessary to associate it with the special action of an accelerator—cardiac nerve, whose functions, hitherto, had not been determined. However this may be, MM. Ludwig and Thiry recognized, as did their predecessors, that irritation of the spinal cord induces modifications in the circulatory phenomena; but instead of admitting that this influence is exercised directly upon the heart, as M. de Bezold had done, they concluded, on the contrary, that it is directed primarily upon the peripheric circulatory system, by means of the vasomotor or vascular nerves to react thence upon the central organ of circulation in an indirect or secondary manner. Such was the status of the subject of the innervation of the heart by the spinal cord, when new experiments, instituted by MM. Cyon and Ludwig

* Ludwig et Thiry, Uber den einflus des Halsmarkes aus den Blutstrom, 1864.

are adduced to corroborate the conclusions preceding, and to develop their consequences. After having admitted, in fact, that the irritation of the spinal cord does not react immediately upon the heart, it remained to explain how the augmentation of sanguineous pressure, which it produces, could result from the direct action upon the peripheric circulatory system.

It is this mechanism that MM. Cyon and Ludwig have demonstrated, by showing that this influence of the spinal cord is transmitted through the mediation of vascular nerves, and especially by the splanchnic vasomotor nerves. Of all the vasomotor nerves of the body, the splanchnic nerves are evidently the most important, and the most capable of modifying the general circulation, by reason of the enormous blood-supply of the splanchnic organs to which they are distributed. M.M. Cyon and Ludwig demonstrate by the aid of accurate experiments, that when the splanchnic nerves are divided, effects are obtained similar to those which result from section of the cord between the occiput and the atlas.

In the two cases, the manometric pressure of the blood diminishes rapidly and considerably in consequence of the paralysis of the vasomotor nerves, and the enlargement of the peripheric vessels which retain the blood in the organs, and effect thus a depletion of the central vascular system. If the peripheric extremities of the divided splanchnic nerves be then irritated, the manometric pressure of the blood is perceived to increase and ascend by reason of the contraction of the splanchnic vessels which drives the blood from the abdomen, where it was accumulated and carried back in a relatively increased quantity, into the cardiac system. Lastly, after section of splanchnic nerves, irritation of the spinal cord separated from the brain, induces no longer, or only to a very insignificant extent, augmentation of the pressure of the blood, because nervous influence can no longer be propagated to the vessels in order to determine their contraction.

After all the preceding facts, it remains well established that the augmentation of the manometric pressure of the blood, could not be the result of the immediate and direct influence of the cord upon the central organ of the circulation; but it would be wrong to come to the same conclusion with regard to the acceleration of the pulsations of the heart, which are observed ordinarily in a manner coincident with the augmentation of the pressure of the blood. Indeed, M. Cyon has proved that these two orders of phenomena can be produced separately, for he has shown that after the section of the splanchnic nerves, when irritation of the spinal cord no longer induces an augmentation of sanguineous pressure, this same irritation still renders apparent the acceleration of the pulsations of the heart solely.

In following up the explanation of this last phenomenon, M. Cyon has even succeeded in establishing clearly that this accelerating influence depends upon an immediate action of the spinal cord upon the heart, and he has demonstrated that it takes places through the intervention of a special cardiac accelerator nerve, which emerges from the spinal column with the third branch of the inferior cervical ganglion.

The direct influence of the spinal cord upon the heart, first indicated by Le Gallois, then recognized by M. de Bezold, actually exists; however, it is necessary to distinguish in the physiological explanation, the fact of the augmentation of the manometric pressure of the blood, from that of the acceleration of the pulsations of the heart.

The augmentation of the sanguineous pressure, results, evidently from the influence of the spinal cord upon the vaso-motor nerves, whilst the acceleration of the pulsations of the heart is, on the contrary, the effect of the direct action of the cord upon the heart itself by the intervention of the special cardiac accelerator nerve. However, if this cardiac nerve, the accelerator of the pulsations of the heart, as well as the splanchnic and vaso-motor nerves, can be, as has already been stated, influenced by mechanical irritation of the spinal cord, it happens likewise, that in the normal or physiological state, these nerves are put into functional activity in an indirect or reflex manner by excitement emanating from the nerves of sensation.

We have already stated at the beginning, that irritation of the nerves of sensibility at the surface of the body, that is to say, irritation of the spinal roots, may react upon sanguineous pressure, and upon the pulsations of the heart. But these reflex actions are even more general, and the new point upon which we wish particularly to direct attention, is that movements take place in the peripheric, or central circulatory system, which are the results of the excitement of the nerves of sensation distributcd to the internal surfrce of the heart. It has been known for a long time that the internal surface of the ventricles of the heart were endowed with sensibility: a member of our commission* had observed that by touching with a thermometer for example, the internal surface of the ventricles in sheep, the pulsations of the heart manifested immediately a great acceleration which could not be explained in this case but by a reflex reaction upon the cardiac accelerator nerve. But, besides this reflex accelerator influence upon the heart, M. Cyon has shown that there exists, still further, a reflex action at once distensive of the peripheral vessels, and depressive of the cardiac circulation, which likewise has for its points of departure excitement of the sensory nerves of the heart.

This important discovery is found detailed and developed in one of the memoirs upon the innervation of the heart presented by M. Cyon at the competition in experimental physiology, entitled "*Upon the reflex action of one of the sensory nerves of the heart upon the motor nerves of the sanguineous vessels.*† In this work, upon which the commission has brought to bear especially its criticism and judgment there is discussed in reality, the discovery of a new sensory nerve of the heart endowed with functions remaining up to this time unknown. We will first examine thc anatomical arrangement of this nerve.

* Claud Bernard, Leçons sur les liquides de l'organisme, t. I, p. 120, 1859

† M. M. E. et M. Cyon ont communiqués a l'Acadamie (25 Mars, 1867), un résumé de leurs recherches sur l'innervation du cœur, exécutés sort a Berlin, dans la laboratoire de M. du Bois Raymond. Soit à Leipsic, avec le concours de M. le professor Ludwig. C'est M. E. Cyon qui a presenté les travaux aux concours de physiologie experimentale, et qui a mis les membres de la commission a meme de rénfeér ses expèriences.

In the rabbit, upon which M. Cyon has particularly experimented, this nerve ordinarily originates by two roots, one of which proceeds from the trunk of the pneumogastric, and the other from the superior laryngeal nerve. Starting from its origin in the superior region of the neck, the sensory cardiac nerve descends parallel to the carotid artery by the side of a filament of the cervical sympathetic, which accompanies, without ever uniting with it.

After having reached the thorax, the sensory cardiac nerve anastomoses with the filaments proceeding from the first thoracic ganglion, and is soon lost in the substance of the heart, or rather in the dense and compact cellular tissue which is situated between the origins of the aorta and the pulmonary artery. In order to experiment upon this nerve, it is laid bare in the living animal, in the middle region of the neck, then it is divided in order to experiment upon the two extremities successively, at the same time that a hæmometer (hæma-dynamometer) is applied to the carotid artery in order to observe the variations which shall supervene in the pressure of the blood. Galvanic irritation of the peripheric, or inferior extremity of this nerve, produces no pain, and remains absolutely without effect upon the manometric pressure of the blood, whilst the galvanic irritation of the superior or central nervous extremity, is on the contrary, painful, and induces in the manometer applied to the carotid artery a considerable depression of five or six centimetres in the column of blood.

This immediate reduction of the pressure of the blood under the influence of the irritation of the central and of the sensory cardiac nerve, is a constant result which has been reproduced under the eyes of the members of this commission; the sanguineous depression coincides exactly with the nervous irritation, and is removed as soon as that ceases. After having established this remarkable reflex of the sensory cardiac nerve upon the blood pressure, it is still necessary to explain its mechanism; it is to this that M. Cyon especially devoted himself. First, upon what organs did this reflex action exert itself? Was it upon the general muscular system, upon the heart or upon the vessels?

In order to eliminate the influence of general movements (which, otherwise, would have augmented the sanguineous pressure instead of diminishing it), the rabbits were paralyzed with curare, which destroys rapidly the properties of the voluntary motor nerves, and permits the persistence, for a long time, of those of the vasomotor nerves, and of the nerves of sensibility, upon animals thus prepared, the irritation of the central extremity of the sensory nerve of the heart, no longer produced any reaction upon the paralyzed limbs, whilst this irritation induced in the manometer the same considerable depression of the blood of 5 or 6 centimetres. It was not any longer upon the heart that the reflex action bore immediately; for after having destroyed all the nerves which return to this organ—irritation of the central extremity of the sensory cardiac nerve still induced diminution in the blood-pressure. Thus we are induced, by the method of exclusion, to suppose that the reflex action ought to bear especially upon the peripheric vascular system; but an induction would not suffice: there was still wanting a direct demonstration, which M. Cyon has given, in pointing out, that when a section of the splanchnic vaso-motor nerve has been previously effected, irritation of the central extremity of the sensory nerve of the heart, no longer produces in the manometer the sanguineous pressure which was previously observed.

Definitely, every experimental analysis which precedes, demonstrates that, in the experience of M. Cyon, irritation of the sensory nerves of the heart reacts exclusively upon the vasomotor nerves in order to produce a depletion of the heart, and, consequently, a diminution of the sanguineous pressure as indicated by the manometer. It is in order to express clearly this constant fact of manometric depression, succeeding irritation of the sensory cardiac filament, that M. Cyon has given this nerve the name of *depressor nerve of the circulation.*

Now, there no longer remains to be made any explanation, for the clear comprehension of the entirely special character of this reflex depressing action, which this sensory nerve of the heart exercises. Physiologists are already acquainted with *direct paralyzing* influences, which, in place of inducing contraction of the muscles, paralyze and relax them. The paralyzing influence of the pneumo-gastric nerve upon the heart, is one of the most conspicuous examples of this singular nervous reaction. Now, at this time, we must admit there are *reflex paralyzing* nervous influences, and the reflex action of the sensory nerve of the heart is precisely of this sort. We have determined, indeed, by direct observation, the paralysis and dilatation of the peripheric arterial vessels at the moment when the sanguineous depression takes place under the influence of the irritation of the sensory nerve of the heart. It is impossible to give, at once, the explanation of these phenomena of nervous paralysis, because they are still involved in many theoretical obscurities, but they are, therefore, less worthy the attention of physiologists, for unexplained facts always contain the germs of the scientific truths of the future.

To recapitulate, the study of the innervation of the heart by the spinal cord has been established of late upon entirely new foundations, thanks to a series of investigations, of which we have believed it our duty to give a rapid epitome in this statement, for the reason that they are all associated, and each is necessary to the understanding of the other.

The discovery of the depressor nerve of the circulation, reveals to us facts of the highest importance, which are destined to throw a vivid and unexpected light upon the problem, hitherto so difficult and so complicated, of the physiology of the nerves of the heart. It has been shown that the heart can, by the aid of the nerves of sensibility with which it is provided, regulate, in some degree, its dimensions according to its necessities, by operating by reflex action upon the general circulation, and we can now comprehend how this perpetual equilibrium, which must exist between the central and the perpetual circulation, is established.

If the sensibility of the walls of the heart be excited by too great sanguineous repletion, there results an energetic reflex action which dilates the capillary vessels, and attracts the blood to the periphery. If, on the contrary, the internal sensibility of the heart is too feebly excited, the peripheral vessels contract and force back the blood toward the centre of circulation.

All the discoveries of M. E. Cyon are conquests by the delicate and difficult method of vivisection. The Academy can not too highly encourage this physiological direction, which alone permits us to carry experimental analysis into complex organisms in order to dissociate phenomena, and to apprehend their essential mechanisms. It is for this reason that the committee has unanimously awarded to M. E. Cyon the prize for experimental physiology for the year 1867.

---

After having regularly awarded the prize, to the work of which we have just now given a report, your committee believe it their duty to ask of the Academy a *second* prize in Physiology, in order to do honor to a series of investigations into the generation and dissemination of intestinal worms, the results of which are recapitulated in a publication by M. Baillet entitled: *Natural history of the intestinal worms of the domestic mammifera.* This work differs altogether from that which precedes it, and, as its name indicates, is a treatise upon Zoology rather than upon Physiology. However, many of the facts in the history of the propagation and migrations of intestinal worms appertain to physiology in this regard, that this history can only be comprehended through an acquaintance with the special properties of the tissues of these creatures, and by the determination, of the condition most especially favorable, in the midst of which these properties of tissues are permitted to be developed.

In order to restrict themselves to the spirit of the competition (concours) they will direct their criticism exclusively upon those portions of the researches of M. Baillet which refer to the embryogenesis and development of intestinal worms.

We will specialize, at first, a group of experiments, in which M. Baillet has studied the influence exercised by the surrounding media upon the development of the eggs and embryogenesis of certain species, at the same time that he has determined the remarkable powers of resistance with which these eggs and embryos are endowed. Whilst adapting them to different temperatures, whilst surrounding them with a liquid alternately pure or corrupt, M. Baillet has seen the segmentation of the yoke arrested, retarded or accelerated, the development of the embryos advance in a progressive manner or suspended, and that, too, upon several repetitions, without, apparently, damaging the embryos. He has thus been able to protract during eleven months, the embryonic development of certain species of ascaris, which, in their normal conditions, according to their temperature, would, in ten days or a month, pass this first phase of their existence.

Other experiments, which associate themselves with the preceding, show us the young ascarides, already formed, remaining stationary, under certain conditions, during a period which might be called indefinite. Mr. Baillet has preserved during nearly two years, under the water, or in wet ground, or simply upon plates of glass, eggs of four species. A. Megalocephala, A. Mystax, A. Suilla, A. Marginata, in which the well-formed embryos are active up to the last day. All these experiments are of a character to prove that the eggs and the embryos of intestinal worms are endowed with a vital tenacity which enables them to resist certain injurious influences of their surrounding medium, and of awaiting, in a latent phase of life, conditions favorable to their development, M. Baillet has rightfully insisted upon these interesting facts. He has been able also to extend his observations upon new species; but he had already been preceded in this path by M. Davaine and M. Leuckart. The first of these authors had determined the property possessed by the eggs of certain intestinal worms of being developed in the dry state; and in relation to the duration of the embryonic development, he had obtained results still more remarkable, for he had preserved in water during five years, eggs of ascarides lumbricoides containing embryos full of life.

M. Baillet has also made experiments with the view to throw light upon the history of *sclerostoma equinum*, and of *strongulus filiaria* of the sheep. It results from these researches that the strongle whilst multiplying on the spot, also propagates itself from one individual to another by means of migration of its embryos. That they may be able to bear the hazards of the route, these are endowed with remarkable vitality. This considerable vital resistance of the embryo of the strongle, compared with the adult worms, had already been remarked in the last century by Camper, upon the strongle of the calf, and M. Davaine, who reports the fact, has first deduced therefrom the consequences which are relative to propation and migration of these worms. But the experiments of M. Baillet are likewise very interesting, in that they have shown that the embryos of strongles can also, although to a less degree than the young ascarides, have the property of remaining stationery in their development, whilst they may not have found the medium for which they were designed.

M. Baillet has further performed numerous experiments upon the cestoids, entering largely upon the path opened by the two savants whose works were honored by the Academy in 1853.

Whilst confirming the general facts, of which we owe the knowledge to MM. Von Siebold, Van Beneden and Kuchenmeister. M. Baillet has been able to fill up a certain number of lacunæ, to resolve several difficulties which the labors of his prodecessors had left in the science, and to refute some errors which tended to propagate themselves, reinforced as they were by great names. But we will not follow the author in the examination of these question, which belong rather to the domain of Zoology than of Physiology.

To recapitulate: although the work of M. Baillet does not include, strictly speaking, physiological discoveries, however it is an important work which has the merit of having confirmed and extended experiments which are of a character to encourage general physiology.

The committee, in honoring the work of M. Baillet, has designed by encouraging zoologists in the experimental study of the tissues of lower animals; and, on the other hand, by recompensing two orders of investigation, performed in totally different directions, it has desired to prove that it comprehends physiological science in its broadest sense, and that it accepts, as belonging to it, all those studies which contribute to the explanation of the phenomena of life. Such is the summary of the motives which have induced the committee to ask for M. Baillet a second prize of Physiology.

The committee has, moreover, directed its attention to a memoir of M. Moura, entitled "The act of deglutition, its mechanism."

The act of deglutition presents a decidedly complex mechanism, which, since the days of Hippocrates, has exercised the sagacity of a large number of physiologists. M. Moura, having at his service laryngoscopic experiment and observation, has assumed in turn the study of this physiological problem, and he has had the merit of adding interesting facts to this subject, already so frequently investigated by skillful experimenters.

From the summary of the researches of M. Moura, it results:

1. That deglutition is effected in a different manner, in man and in the dog.

2. As to the deglutition in man, the three periods assigned to the act should be reduced to two. According to M. Moura, deglutition really commenced only when the alimentary matters disseminated upon the tongue have arrived at the free edge of the epiglottis. From which it results that the passage of the food across the isthmus of the fauces is the last phenomenon of mastication, and does not really belong to the act of deglutition. During this passage only the lower third of the epiglottis closes the larynx; whilst the upper two-thirds remain elevated, and unite with the larynx in forming an orifice, and an irregular conduit into which the bolus is pushed back by the base of the tongue.

3. Fluids are collected into the same channel as the food, and are not introduced into the pharynx by passing over the sides of the epiglottis.

The committee accords to M. Moura an honorable mention for his experimental investigations into the phenomena of deglutition.

In conclusion, the committee of the Concours of Experimental Physiology, for the year 1867, decree the prize for experimental physiology to M. E. Cyon, for his work upon *the innervation of the heart by the spinal cord.* It asks of the Academy a second prize of experimental physiology to honor the investigations of M. Baillet upon the generation of intestinal worms in domestic animals. And it awards an honorable mention to M. Moura for his work on deglutition.

The Academy adopts the propositions of the committee.

